# Heme oxygenase-1 and malaria pathogenesis

**DOI:** 10.3389/fimmu.2026.1777520

**Published:** 2026-02-27

**Authors:** Theophilus Wakai, Shalom Chinedu, Israel Afolabi

**Affiliations:** 1 Malaria Research Unit, Covenant Applied Informatics and Communication-Africa Centre of Excellence (CApIC-ACE), Ota, Nigeria; 2 Department of Biochemistry, College of Science and Technology, Covenant University, Ota, Nigeria; 3 Covenant University Public Health and Wellbeing Research Cluster (CUPHWERC), Covenant University, Ota, Nigeria

**Keywords:** biomarker, heme oxygenase-1, malaria, oxidative stress, pathogenesis

## Abstract

Malaria is a life-threatening parasitic disease and remains a major cause of morbidity and mortality worldwide. The clinical course of malaria ranges from uncomplicated infection to severe disease, driven by extensive hemolysis, inflammation, and oxidative stress. Heme oxygenase-1 (HO-1), an inducible enzyme involved in heme degradation, has been demonstrated to play a crucial role in the host’s response to *Plasmodium* infection. Experimental and clinical studies suggest that HO-1 is strongly induced during malaria and plays a crucial role in regulating inflammation, oxidative damage, and tissue injury. In murine models, HO-1 induction confers protection against severe malaria complications, including cerebral malaria and organ dysfunction, partly by modulating pro-inflammatory cytokines and vascular permeability. Conversely, elevated HO-1 expression in specific immune cell populations has been associated with heightened inflammatory responses and disease severity in humans, highlighting its context-dependent effects. Here, we review the key roles of HO-1 in malaria pathogenesis, emphasising its dual protective and pathological functions, and discuss its potential relevance as a diagnostic and prognostic biomarker and as a therapeutic target.

## 1 Introduction

Malaria disease, a life-threatening parasitic infection, remains a major global health challenge, particularly in sub-Saharan Africa, Southeast Asia, and Latin America (1). It is caused by protozoan parasites of the genus *Plasmodium*, which are transmitted to humans through the bites of infected female *Anopheles* mosquitoes. Five *Plasmodium* species are known to infect humans, including *P. falciparum, P. vivax, P. malariae, P. ovale, and P. knowlesi*, with marked differences in their clinical manifestations and disease severity (2, 3).

*Plasmodium falciparum* is the most virulent and prevalent species in Africa, responsible for the vast majority of severe malaria disease cases and deaths. Infections with *P. falciparum* can rapidly progress to severe complications such as cerebral malaria, severe anaemia, acute kidney injury, and acute respiratory distress syndrome, often leading to fatal outcomes if not promptly treated (4, 5). In contrast, *Plasmodium vivax* is the most geographically widespread species outside of Africa and is characterised by its ability to form dormant liver stages (hypnozoites), leading to relapsing infections that contribute significantly to morbidity and disease transmission (6, 7). Although generally considered less severe than *P. falciparum*, *P. vivax* can also cause severe malaria disease, including severe anaemia and respiratory distress, especially in vulnerable populations (8–11). *Plasmodium ovale* and *Plasmodium malariae* cause chronic, generally milder forms of malaria disease, though *P. malariae*can lead to nephrotic syndrome (12, 13). More recently, *Plasmodium knowlesi*, a simian malaria parasite, has emerged as a main cause of human malaria disease in Southeast Asia, capable of causing rapidly progressive and severe infections (13). Knowledge of the distinct pathogenic and host-response mechanisms elicited by each Plasmodium species is essential for standardising effective diagnostic, therapeutic, and preventive strategies against malaria. Awareness campaigns are necessary to improve the knowledge and practice of malaria control efforts. Though higher knowledge does not always translate into full practice behaviours, context-specific campaigns require improvement (14).

Accurate and timely diagnosis is paramount for effective malaria management, enabling prompt treatment, preventing severe complications, and curbing transmission (7). Despite significant advancements, several challenges persist in malaria diagnostics, particularly in resource-limited settings where the disease burden is highest (11, 15, 16). The current diagnostic landscape for malaria primarily relies on three main approaches: microscopy, rapid diagnostic tests (RDTs), and molecular methods (17, 18).

While microscopy and RDTs remain the most widely used diagnostic tools in endemic regions due to cost and accessibility, their sensitivity declines at low parasitemia compared to PCR-based methods. Microscopy, the gold standard for over a century, involves the microscopic examination of Giemsa-stained blood smears to identify and quantify *Plasmodium* parasites (19, 20). In clinical settings, skilled microscopists can differentiate *Plasmodium* species, stage parasites, and estimate parasite density, which is critical for guiding treatment decisions. In 2022, microscopy was used for approximately 25% of malaria diagnostic tests globally (21). While highly sensitive (detecting as few as 50–100 parasites/µL) and specific when performed by experienced personnel, its effectiveness depends heavily on operator skill, reagent quality, and equipment availability. It is also labour-intensive and time-consuming, making it less suitable for mass screening or remote areas lacking trained personnel (18). Rapid Diagnostic Tests (RDTs) have revolutionised malaria diagnosis, especially in peripheral health facilities and community settings, due to their simplicity, speed, and independence from electricity or specialised equipment (22, 23). RDTs detect specific parasite antigens, most commonly *P. falciparum* histidine-rich protein 2 (HRP2) and/or *P. vivax* lactate dehydrogenase (pLDH), in a drop of blood. In 2022, RDTs accounted for approximately 70% of malaria diagnostic tests globally (24). WHO-prequalified RDTs typically demonstrate sensitivities exceeding 95% for *P. falciparum* at parasite densities above 1000 parasites/µL, though sensitivity can drop at lower parasite densities or in cases of HRP2 gene deletions (25). Their ease of use has significantly expanded access to diagnosis. Still, limitations include the inability to quantify parasite density, persistence of HRP2 antigen after parasite clearance (leading to false positives), and reduced sensitivity for non-*falciparum* species (26).

To enhance accuracy and detect low-density infections, molecular diagnostic methods, primarily Polymerase Chain Reaction (PCR), are increasingly employed, particularly in research settings, for surveillance and to confirm microscopy or RDT results (27, 28). PCR-based assays amplify parasite-specific DNA or RNA sequences, offering very high sensitivity (detecting as few as 1–5 parasites/µL) and specificity, enabling precise species identification and detection of mixed infections (29). While invaluable for epidemiological studies and confirming cryptic infections, PCR requires sophisticated laboratory infrastructure, specialised equipment, and trained personnel, making it less feasible for routine point-of-care diagnosis in many endemic areas (19).

These established diagnostic tools, while foundational, face ongoing challenges related to their operational characteristics, cost-effectiveness, and adaptability to evolving epidemiological landscapes (30, 31). These emerging technologies often leverage advances in molecular biology, immunology, and microfluidics to overcome the limitations of current methods, aiming to enhance sensitivity, specificity, and user-friendliness at the point of care (22). Notably, most conventional diagnostics focus primarily on parasite detection and burden, offering limited insight into host-mediated pathological processes that drive disease severity (32). This gap has stimulated interest in host-derived biomarkers that reflect hemolysis, oxidative stress, and inflammation, which are the central processes in malaria disease pathogenesis (33). The plasmodium species-specific differences in parasitemia, hemolysis, and inflammatory burden are central to understanding host stress responses, including the differential induction of heme oxygenase-1. Generally speaking, while microscopy and RDTs remain indispensable in endemic settings due to cost and accessibility, their reduced sensitivity at low parasitemia underscores the need for adjunct host-based biomarkers that better reflect disease severity.

In recent years, heme oxygenase-1 (HO-1) has gained attention as a potential adjunct biomarker that captures the host’s physiological response to malaria-induced hemolysis and tissue injury, complementing existing parasite-based diagnostic approaches (34).

Despite biological differences among *Plasmodium* species, the hallmarks defining their pathogenesis are similar. One major mechanism is the extensive destruction of red blood cells during the erythrocytic stage of infection (9) (35). Following invasion of erythrocytes, *Plasmodium* parasites rely on host haemoglobin as their primary nutrient source (36). Haemoglobin digestion occurs within the parasite’s digestive vacuole and leads to the liberation of large quantities of free heme, a highly reactive iron-containing molecule (37).

Under normal physiological conditions, heme is tightly sequestered within haemoglobin; however, during malaria infection, parasite-mediated haemoglobin digestion combined with immune-mediated and non-immune hemolysis results in the accumulation of free heme in the circulation (38, 39). Free heme is intrinsically cytotoxic due to its ability to catalyse the formation of reactive oxygen species, promote lipid peroxidation, and disrupt cellular membranes (40). In addition to its oxidative effects, free heme functions as a potent pro-inflammatory danger signal, activating endothelial cells and innate immune pathways, thereby contributing to vascular dysfunction and tissue injury (36, 41).

Excess circulating heme has been implicated in several pathological features of malaria, including anemia, endothelial activation, microvascular obstruction, and organ-specific complications such as acute kidney injury and cerebral malaria (9, 36). These effects are particularly pronounced in severe malaria, where parasite biomass and hemolysis are markedly increased (42, 43),

To counteract the toxic effects of free heme, the host activates tightly regulated heme-detoxification pathways. Central to this response is heme oxygenase-1 (HO-1), an inducible stress-response enzyme that catalyses the degradation of heme into biliverdin, carbon monoxide, and free iron (44, 45). Through these actions, HO-1 exerts antioxidant, anti-inflammatory, and cytoprotective effects, thereby limiting heme-mediated tissue damage (46–48). As a consequence, the efficiency of host heme-handling mechanisms plays a critical role in shaping malaria severity and clinical outcome (49).

## 2 Biochemical and physiological role of heme oxygenase

Heme oxygenase-1 (HO-1) is an inducible, stress-responsive enzyme that catalyses the degradation of free heme into biliverdin, carbon monoxide (CO), and ferrous iron (Fe²^+^) (50). Biliverdin is subsequently converted to bilirubin, a potent endogenous antioxidant, while CO functions as a signalling molecule with anti-inflammatory, anti-apoptotic, and vasoregulatory properties (51). Beyond its metabolic role, HO-1 has emerged as a critical regulator of host responses in infectious, inflammatory, and malignant diseases (52, 53). In the context of malaria, HO-1 expression is strongly induced by hemolysis-driven oxidative stress and inflammation, positioning it at the intersection of host defence, tissue protection, and immune regulation (38, 54). The heme oxygenase (HO) system is a critical enzymatic pathway that catabolizes heme, a prosthetic group essential for numerous biological processes but also highly toxic when free in cells (50). This system plays a fundamental role in cellular homeostasis, responding to various stressors, including oxidative stress, inflammation, and hypoxia (46, 49).

### 2.1 Components and function of the heme oxygenase system

The HO system comprises three distinct isoforms: heme oxygenase-1 (HO-1), heme oxygenase-2 (HO-2), and heme oxygenase-3 (HO-3) (55). Also known as heat shock protein 32 (HSP32), HO-1 is the inducible isoform (56, 57). Its expression is rapidly upregulated by a wide array of stimuli, including heme, heavy metals, cytokines, hypoxia, and oxidative stress (55, 58). This inducible nature underscores its role as a crucial cytoprotective enzyme, particularly in conditions of cellular stress and inflammation, such as those encountered during malaria disease. In contrast to HO-1, HO-2 is constitutively expressed in various tissues, particularly in the brain, testes, and endothelium, and its expression is largely unaffected by stress stimuli. HO-2 is thought to play a role in maintaining basal heme homeostasis and may have specific functions as a gasotransmitter, given its presence in neuronal tissues (47, 55). The existence and functional significance of HO-3 are less clearly defined. It was initially identified as a distinct gene product but is now often considered a pseudogene or a variant of HO-2 in humans, with limited enzymatic activity (59). Unlike HO-2, which is constitutively expressed, HO-1 is strongly inducible, and in malaria disease, its upregulation is directly driven by parasite-induced hemolysis, which releases large amounts of free heme, a potent stimulus for HO-1 expression. For the purpose of understanding its role in malaria disease, HO-1 is the primary focus due to its inducible nature and potent cytoprotective functions.

The enzymatic reaction catalysed by HO involves the oxidative cleavage of the heme molecule (iron protoporphyrin IX) at the α-methene bridge (60). This process requires molecular oxygen and NADPH-cytochrome P450 reductase as an electron donor. The breakdown of one heme molecule yields three key products:

Biliverdin, a green pigment that is rapidly reduced by biliverdin reductase to bilirubin, a yellow pigment. Both biliverdin and bilirubin are potent antioxidants, capable of scavenging reactive oxygen species (ROS) and reactive nitrogen species (RNS) (45, 51).

**Figure 1:**
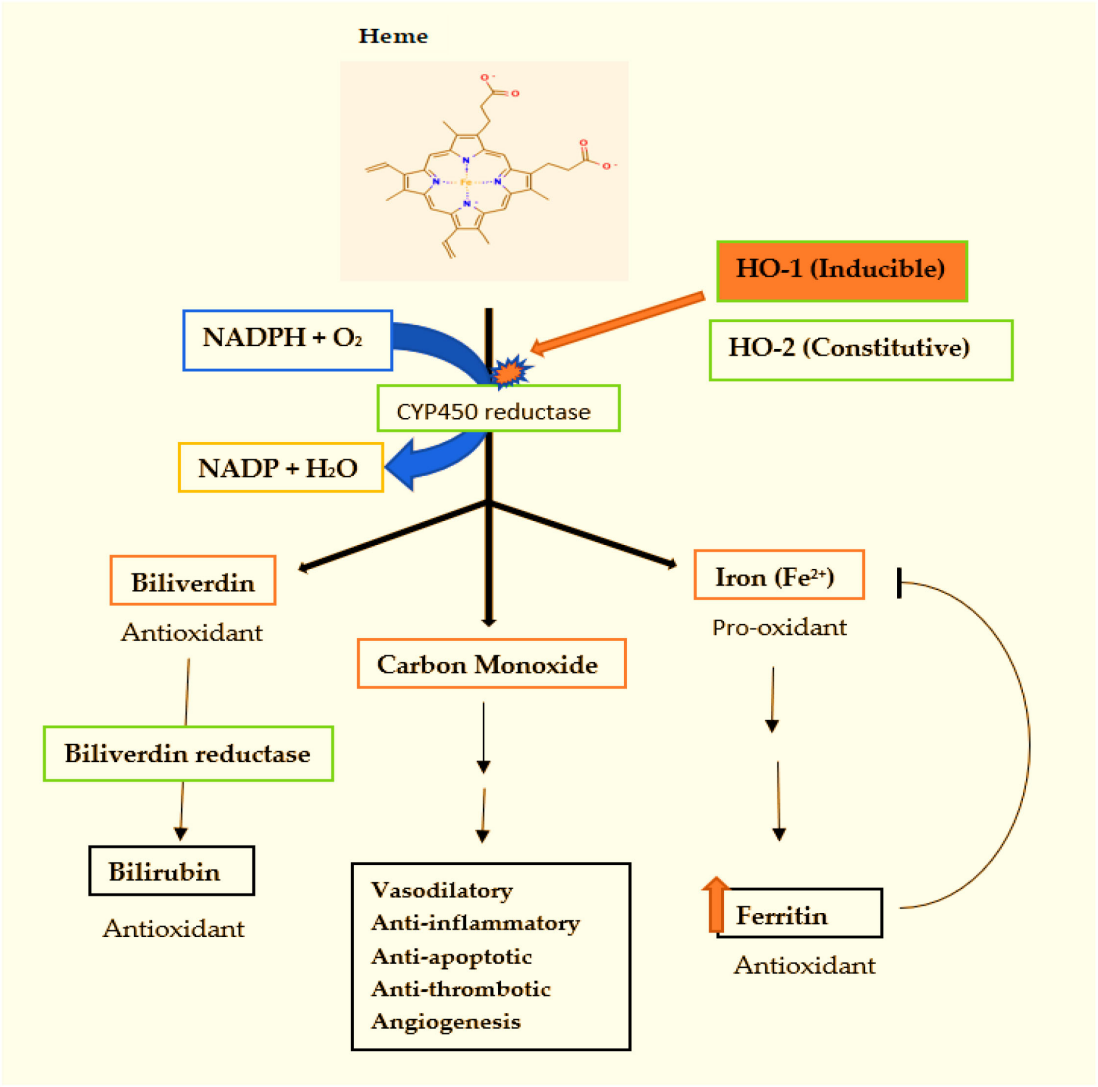
Schematic representation of the heme oxygenase pathway and its cytoprotective byproducts, Illustration by TNW, created using Microsoft Word. HO-1, heme oxygenase-1; HO-2, heme oxygenase-2; NADPH, nicotinamide adenine dinucleotide phosphate; CO, carbon monoxide; Fe²^+^, ferrous iron.

Carbon Monoxide (CO), a gaseous molecule that acts as a signalling molecule (gasotransmitter) with diverse physiological effects, including vasodilation, anti-inflammatory actions, and anti-apoptotic properties (61). Free iron (Fe²^+^) released from heme is a potent pro-oxidant, but HO-1 induction promotes ferritin upregulation to sequester this iron, preventing harmful Fenton reactions and limiting pathogen access (36, 62). The molecular and physiological processes of Heme metabolism are schematically illustrated in **Figure 1**.

Free heme is degraded by heme oxygenase enzymes in the presence of oxygen, NADPH, and cytochrome P450 reductase (1). Heme oxygenase-1 (HO-1) is inducible, whereas heme oxygenase-2 (HO-2) is constitutively expressed. Heme degradation generates biliverdin, carbon monoxide (CO), and ferrous iron (Fe²^+^). Biliverdin is subsequently converted to bilirubin by biliverdin reductase, and both metabolites exert antioxidant effects. Carbon monoxide mediates vasodilatory, anti-inflammatory, anti-apoptotic, and anti-thrombotic effects. Released iron, which is potentially pro-oxidant, is sequestered in ferritin, thereby limiting oxidative damage.

### 2.2 The double-edged sword of heme oxygenase in malaria disease

Heme oxygenase-1 (HO-1), an inducible enzyme, plays a complex and often paradoxical role in the host response to malaria disease. Its activity, and the subsequent production of its byproducts—carbon monoxide (CO), biliverdin (subsequently reduced to bilirubin), and free iron—can exert both protective and detrimental effects on the host, thus representing a “double-edged sword” in the context of *Plasmodium* infection (38, 44, 63). HO-1 serves a dual purpose in the pathogenesis of malaria. First, its enzymatic activity contributes to cellular protection by detoxifying heme, thereby preventing oxidative damage and reducing local inflammation. Second, HO-1-derived products, including carbon monoxide, exhibit immunomodulatory properties that influence host-pathogen interactions (64). For instance, the upregulation of HO-1 has been associated with protection against severe malaria syndromes, such as cerebral malaria (65), through suppression of hyperinflammatory responses and amelioration of endothelial dysfunction (66, 67). Conversely, excessive HO-1 induction can impair granulocyte mobilisation, hinder immunity against secondary bacterial infections, and contribute to disease progression in certain contexts, such as malaria-associated sepsis (1, 2). The net clinical outcome depends on the infection stage, parasite burden, host genetic background (specifically, HMOX1 polymorphisms), and the presence of co-infections. This duality explains contradictory findings in clinical studies and highlights the need for personalised therapeutic approaches.

Accumulating experimental and clinical evidence suggests that HO-1 exerts context-dependent effects during malaria disease. While early or localized HO-1 induction confers protection against severe malaria complications—such as cerebral malaria (43) and organ damage—through mitigation of oxidative injury and modulation of inflammatory responses, excessive or sustained HO-1 expression has been associated with impaired immune function, increased susceptibility to secondary infections, and severe disease outcomes in humans (38, 65, 67). This duality highlights HO-1 as a molecular “double-edged sword” in malaria pathogenesis (63). The induction of HO-1 is generally considered a crucial component of the host’s innate immune response to oxidative stress and inflammation, both hallmarks of malaria disease. The role of HO-1 in malaria disease is highly context-dependent, influenced by infection stage, parasite species, host genetic factors, and disease severity. A finely tuned regulation of HO-1 activity is critical for host survival, where appropriate induction can confer protection, while dysregulation may contribute to pathology (1). Overall, this context-dependent balance between the protective and potentially deleterious effects of HO-1 directly influences malaria disease severity and survival outcomes. These contrasting effects highlight the dual, context-dependent role of HO-1 in malaria disease. Thus, HO-1 functions as a double-edged sword in malaria disease, conferring cytoprotection under controlled induction while potentially impairing antiparasitic immunity when excessively or persistently expressed, as summarized in **Table 1**.

**Table 1:** Overview of the key studies of HO-1 in malaria disease pathogenesis and clinical implications.

Context	Protective mechanisms	Pathological mechanisms	Clinical implications	Key references
Oxidative Stress	Degradation of pro-oxidant free heme; generation of antioxidant bilirubin; prevention of lipid peroxidation	Iron release may exacerbate oxidative damage if ferritin capacity is exceeded; chronic activation may deplete cellular resources	Therapeutic HO-1 induction may benefit acute severe malaria but may pose risks during chronic infection	(43, 68, 69)
Inflammation	Carbon monoxide (CO) suppresses TNF-α, IL-1β, and IL-6; promotes M2 macrophage polarization; reduces endothelial activation	Excessive anti-inflammatory effects may impair parasite clearance; increased susceptibility to secondary bacterial infections	Timing of HO-1 modulation is critical; early induction may be protective, whereas late induction may hinder immune control	(35, 44, 66, 70)
Cerebral Malaria	Maintenance of blood–brain barrier integrity; reduction of neuroinflammation; prevention of hemorrhagic damage	Elevated plasma HO-1 associated with severe disease, possibly reflecting tissue injury rather than causation	HO-1 levels correlate with disease severity; distinction between biomarker and therapeutic target remains unclear	(66, 70, 71)
Hemolytic Anemia	Accelerated heme clearance; prevention of heme-mediated vascular damage; support of erythropoiesis via iron recycling	Excessive iron sequestration may contribute to hypoferremia; bilirubin accumulation may increase the risk of jaundice	Monitoring of bilirubin and iron status is required; HO-1 induction may alleviate hemolysis but necessitates iron management	(72, 73)
Renal Dysfunction	Protection of renal tubular epithelium; reduction of ischemic injury; prevention of heme-cast nephropathy	Iron deposition in renal tubules if ferritin saturation occurs; CO may affect renal blood-flow autoregulation	HMOX1 long alleles are associated with protection against malaria-associated acute kidney injury	(74, 75)
Immune Regulation	Limitations of excessive T-cell activation: prevention of cytokine storm; reduction of complement activation	Impaired neutrophil recruitment; reduced bacterial killing capacity; delayed parasite clearance	Balanced HO-1 activity is required; excessive suppression increases the risk of sepsis in co-infected patients	(35, 76)

### 2.3 Relevance of the Ho-1 to malaria disease

**Figure 2:**
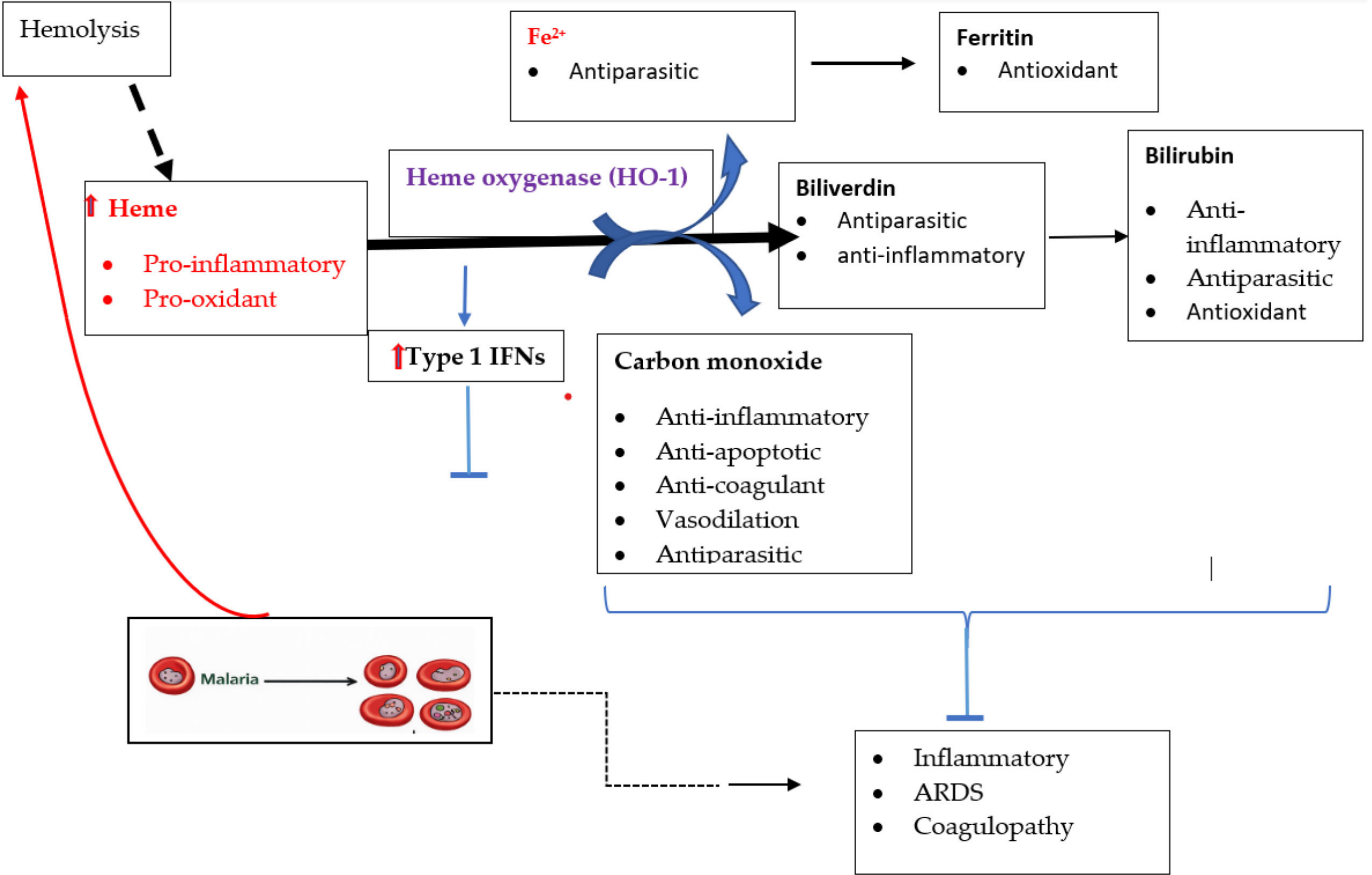
Role of heme oxygenase-1 (HO-1) in modulating hemolysis-driven inflammation during malaria infection. Illustration by TNW, created using shape tools in Microsoft Word.

During a malaria attack, the massive breakdown of haemoglobin within infected red blood cells by *Plasmodium* parasites leads to a significant release of free heme, which is highly toxic to host cells (38). This heme overload, coupled with the intense inflammatory response and oxidative stress characteristic of malaria, strongly induces HO-1 expression in various host tissues (56). The induction of HO-1 is thus a critical host defence mechanism that detoxifies free heme and mitigates the associated cellular damage and inflammation. Biomarkers currently used as prognostic markers of malaria severity include parasitemia levels, lactate dehydrogenase, and inflammatory cytokines (77, 78). The limitations of current tools include delayed detection, inability to differentiate between malaria species, and poor prognostic accuracy for severe cases (19). There is a pressing need for biomarkers that can be measured non-invasively, provide rapid results, and correlate with disease progression. Emerging diagnostic technologies, including molecular and imaging-based methods, have incorporated the detection of hemozoin, a crystalline byproduct of haemoglobin digestion, as a malaria biomarker (79). Given the close interplay between hemozoin formation and host heme metabolism, HO-1 activity may serve as a complementary diagnostic and pathophysiological marker alongside conventional tools such as rapid diagnostic tests (RDTs) and PCR (80). The pathways illustrated in **Figure 2** provide the mechanistic framework detailing the biochemical activity of HO-1, its immunomodulatory effects, and its protective roles during malaria infection.

Malaria-induced hemolysis releases free heme, which promotes oxidative stress and inflammation and induces heme oxygenase-1 (HO-1). HO-1 degrades heme into Fe²^+^, biliverdin/bilirubin, and carbon monoxide, which exert antioxidant, anti-inflammatory, antiparasitic, and cytoprotective effects. HO-1 activity is also associated with type I interferon induction, collectively limiting inflammation, ARDS, and coagulopathy in severe malaria.

### 2.4 Unique aspects of HO-1 in malaria pathogenesis

Aside from its well-established cytoprotective function, HO-1 has been shown to exhibit unique interactions within the complex pathophysiology of malaria disease. For example, HO-1 has been reported as a notable regulator of microvascular integrity, particularly in severe forms such as cerebral malaria (81). Prior studies have highlighted that HO-1 induction, and specifically the production of CO, can preserve the integrity of the blood-brain barrier (BBB) (48, 82). For instance, in experimental cerebral malaria (ECM) models, pharmacological induction of HO-1 or administration of CO has been shown to reduce BBB breakdown, leukocyte sequestration in brain microvessels, and ultimately improve survival (1, 2). This protective effect is thought to be mediated by CO’s ability to modulate endothelial cell function, reduce adhesion molecule expression, and promote vasodilation, thereby counteracting the microvascular obstruction and inflammation that are central to cerebral malaria pathology (42, 83). Furthermore, HO-1 has been implicated in modulating the adherence of *P. falciparum*-infected red blood cells (iRBCs) to endothelial cells, a key event in malaria pathogenesis (84, 85). While the precise mechanisms remain under investigation, some evidence suggests that HO-1-derived products may influence endothelial receptor expression or iRBC cytoadherence properties, thereby impacting sequestration and disease severity (86, 87). By preserving microvascular integrity and limiting parasite sequestration, these mechanisms reduce the risk of cerebral complications and improve survival in severe malaria disease.

### 2.5 HO-1 modulation of immune function in malaria disease

Heme oxygenase-1 (HO-1) is a potent immunomodulatory enzyme whose induction profoundly influences both innate and adaptive immune responses during malaria infection. Beyond its role in heme detoxification, HO-1 acts as a critical regulator of inflammation, immune cell function, and immune homeostasis, thereby shaping disease outcomes (50, 56). One of the most extensively described functions of HO-1 in malaria is its anti-inflammatory effect. HO-1 and its metabolic by-products—carbon monoxide (CO) and bilirubin—suppress the production of pro-inflammatory cytokines, including tumour necrosis factor-alpha (TNF-α), interleukin-1β (IL-1β), and interleukin-6 (IL-6), which are central mediators of immunopathology in severe malaria (88, 89). Excessive production of these cytokines contributes to endothelial activation, vascular leakage, and tissue damage (86, 90). In experimental *Plasmodium berghei* ANKA infection, HO-1 induction has been shown to attenuate TNF-α levels and dampen the inflammatory cascade responsible for the development of experimental cerebral malaria (ECM) (91, 92) thereby improving survival outcomes. In parallel, HO-1 promotes the expression of anti-inflammatory cytokines, such as interleukin-10 (IL-10) (46, 93) which plays a critical role in limiting immune-mediated tissue injury and maintaining immune balance during infection.

HO-1 also exerts cell-specific immunomodulatory effects, particularly on macrophages and T lymphocytes. In macrophages, HO-1 induction favours polarisation toward an M2-like phenotype, characterised by enhanced phagocytic capacity, tissue repair functions, and reduced secretion of pro-inflammatory mediators (67, 94, 95). This shift contributes to resolving inflammation and protecting against oxidative stress-induced tissue damage. In contrast, HO-1 activity in T cells has been associated with suppressed proliferation and reduced effector cytokine production, suggesting a role in limiting excessive T cell–driven inflammation (56, 96). While this immunoregulatory effect may be protective by preventing immunopathology, it can also be detrimental by impairing effective anti-parasitic immunity. Indeed, it has been demonstrated that HO-1 induction under certain conditions compromises malaria-specific T cell responses, potentially delaying parasite clearance and promoting persistent infection (97, 98).

Furthermore, HO-1 influences dendritic cell (DC) maturation and antigen presentation, thereby indirectly modulating adaptive immune activation. Elevated HO-1 expression in DCs has been shown to inhibit their maturation, reduce expression of co-stimulatory molecules, and impair antigen-presenting capacity (99, 100). This results in diminished T cell priming and activation, which may contribute to either parasite immune evasion or the prevention of overwhelming inflammatory responses, depending on the disease context and the timing of HO-1 induction (101, 102). A hypothesis is that HO-1-like molecules may be implicated in immune dysregulation by accelerating telomere shortening in immune cells during malaria (103). However, studies on the impact of malaria and other infections on this ageing hallmark are scarce (104). It is important to recognise that while the immunosuppressive and anti-inflammatory properties of heme oxygenase-1 (HO-1) are essential for limiting immune-mediated pathology during malaria infection, excessive or prolonged HO-1 activity may compromise the protective immune responses required for efficient parasite clearance. Consequently, the immunomodulatory functions of HO-1 operate as a finely balanced regulatory mechanism that critically influences malaria disease severity and clinical outcome (67). Together, these immunomodulatory effects help limit immune-mediated tissue damage while shaping malaria disease severity and overall clinical outcome.

### 2.6 Organ-specific protection of HO-1 in malaria disease

The cytoprotective and immunomodulatory functions of heme oxygenase-1 (HO-1) are especially important in the context of organ-specific damage caused by severe malaria. Beyond its systemic anti-inflammatory effects, HO-1 acts locally within vulnerable tissues to limit oxidative injury, maintain microvascular function, and reduce immune-mediated pathology (88, 105). These protective actions have direct clinical relevance, as organ failure remains a major determinant of mortality in malaria patients. Some of the organ systems protected include: neurological, renal, cardiovascular, pulmonary and respiratory systems:

In cerebral malaria, HO-1 has emerged as a key protective factor against the cascade of events leading to neurological injury and death. Experimental studies demonstrate that HO-1 induction preserves blood–brain barrier integrity, limits excessive neuroinflammation, and reduces microvascular obstruction caused by sequestration of parasitised erythrocytes and activated immune cells (48, 92). By mitigating these pathological processes, HO-1 reduces cerebral edema, neuronal damage, and mortality. From a clinical standpoint, this is particularly significant, as neurological sequelae and high case-fatality rates remain hallmarks of cerebral malaria, especially among children (81). Therapeutic strategies that enhance HO-1 activity may therefore hold promise in reducing both acute mortality and long-term neurological impairment (106).

Severe malarial anaemia represents another major life-threatening complication, driven largely by extensive intravascular hemolysis and the consequent release of free heme. Free heme is highly toxic, promoting oxidative stress, inflammation, and damage to erythroid progenitor cells (36, 49). HO-1 plays a central role in counteracting these effects by degrading free heme and facilitating iron sequestration through ferritin, thereby limiting heme-mediated cytotoxicity (88). Through this mechanism, HO-1 protects the bone marrow microenvironment and supports the recovery of effective erythropoiesis. This protective role is particularly relevant in pediatric malaria, where severe anaemia is a leading cause of hospitalisation and death.

Malaria-associated acute kidney injury (AKI) is increasingly recognised as a serious complication of severe disease and is associated with poor prognosis (107). The pathogenesis of AKI in malaria involves hemolysis-induced oxidative stress, systemic inflammation, endothelial dysfunction, and impaired renal microcirculation. HO-1 has been widely shown to confer renal protection in diverse models of kidney injury through its antioxidant and anti-inflammatory actions (74, 107, 108). In malaria, induction of HO-1 in renal tubular and endothelial cells may attenuate heme toxicity, reduce inflammatory damage, and preserve renal function (109), 110). These effects are clinically relevant, as early renal protection could significantly improve survival and reduce the need for renal replacement therapy in severe cases.

Pulmonary involvement, including acute respiratory distress syndrome (ARDS), is another severe manifestation of malaria that carries a high risk of mortality. HO-1 contributes to pulmonary protection by suppressing inflammatory responses, enhancing endothelial barrier integrity, and improving microvascular perfusion, effects that are partially mediated by carbon monoxide signalling (38, 42). The lungs are constantly exposed to numerous toxins in the air we breathe, so they rely heavily on HO-1 for protection. HO-1 plays a crucial role in mitigating oxidative damage during lung inflammation, acute respiratory distress syndrome (ARDS), and other conditions induced by environmental stressors, such as particulate matter (111–113). By limiting endothelial cell apoptosis and vascular leakage, HO-1 may help prevent progression to respiratory failure in patients with severe malaria, underscoring its role in protecting lung function during systemic infection.

Malaria has been reported to accelerate cellular ageing by shortening telomere length (103), but this effect can be reversed after treatment (114). Heme oxygenase-1 (HO-1) has been shown to protect cells from molecular hallmarks that would otherwise accelerate cellular ageing. Notably, a 14-kDa splice variant of HO-1 has been reported to promote cell proliferation, suggesting a role for HO-1 in maintaining cellular replicative capacity (115). This effect may be linked, at least in part, to the modulation of STAT1-dependent signalling pathways that are upregulated during inflammatory states, such as those observed in malaria disease states (116). Although the direct regulation of telomeres by HO-1 via STAT1 has not been conclusively demonstrated, accumulating evidence supports an indirect HO-1–STAT1–hTERT axis, in which HO-1 attenuates cytokine-driven STAT1 activation, thereby relieving telomerase repression and mitigating inflammation-associated telomere attrition (117). Exactly, the mechanisms driving these have not been reported, although studies suggest that cytokines such as IL-6 and IFN-γ could be implicated (118, 119). Historically, research investigating the factors associated with cellular ageing in the context of infections has primarily focused on viral infections. This phenomenon was surprisingly neglected in the context of malaria (103, 104). The evidence showcases HO-1’s capacity to provide organ-specific protection and its relevance not only as a molecular marker of disease severity but also as a potential therapeutic target (52, 120, 121). While excessive or dysregulated HO-1 activity may impair parasite clearance, appropriately timed induction of HO-1 could help to limit organ damage and improve clinical outcomes when used as an adjunct to antimalarial therapy. Translating these insights from experimental models into clinical practice remains a challenge, but the growing body of evidence positions HO-1 as a critical mediator linking host tolerance, immune regulation, and survival in malaria (67). These organ-protective effects collectively reduce neurological injury, renal failure, respiratory distress, and mortality in severe malaria.

## 3 Clinical relevance and biomarker potential of HO-1 in malaria disease

The intricate roles of HO-1 in malaria pathogenesis and host defence suggest its significant clinical relevance, both as a potential therapeutic target and as a biomarker of disease severity and prognosis (122, 123). However, its utility is often modulated by the specific *Plasmodium* species involved and the broader context of the host’s physiological state.

### 3.1 Species-dependent clinical relevance

The clinical relevance of HO-1 in malaria disease can vary significantly across different *Plasmodium* species, reflecting their distinct pathogenic mechanisms and host-parasite interactions.

#### 3.1.1 *P. falciparum* malaria

Given that *P. falciparum* is responsible for the most severe forms of malaria, including cerebral malaria, severe anaemia, and multi-organ failure, much of the research on HO-1’s clinical relevance has focused on this species (4). In *P. falciparum* infections, high levels of HO-1 induction are often observed, particularly in severe cases (35). This robust induction is likely a host-protective response to the massive heme release and oxidative stress induced by *P. falciparum*’s rapid replication and high parasite biomass (124). Studies have shown that higher HO-1 expression or activity is associated with better outcomes in patients with severe *P. falciparum* malaria, particularly in those with cerebral malaria, suggesting a protective role (66, 82). However, in some contexts, excessive or dysregulated HO-1 activity might also contribute to immunosuppression, potentially hindering adequate parasite clearance, though this remains an area of active investigation (125).

#### 3.1.2 *P. vivax* malaria

While *P. vivax* generally causes milder disease than *P. falciparum*, it can also lead to severe manifestations, including severe anaemia and respiratory distress (6, 9). The clinical relevance of HO-1 in *P. vivax* malaria is less extensively studied but is emerging. *P. vivax* also causes significant hemolysis, leading to heme overload and oxidative stress, which would theoretically induce HO-1 (9, 126). Given the propensity for relapses in *P. vivax* infections, the role of HO-1 in chronic inflammation and host immune responses to repeated parasitic challenges could be vital. Further research is needed to delineate the specific impact of HO-1 on *P. vivax* pathogenesis and host immunity, especially concerning its role in preventing severe anaemia and managing chronic inflammation.

#### 3.1.3 *P. knowlesi* malaria

*P. knowlesi* can cause rapidly progressive and severe disease, often leading to high parasite densities and significant hemolysis (13). The acute and severe nature of *P. knowlesi* infections suggests that HO-1 induction would be a critical host response to the intense oxidative stress and heme toxicity. Understanding HO-1’s role in *P. knowlesi* infections could provide insights into managing the rapid progression to severe disease and associated organ damage (4, 67).

### 3.2 HO-1 as a biomarker for malaria disease

The inducible nature of heme HO-1 and its central role in counteracting heme-driven oxidative stress and inflammation have attracted considerable attention across infectious diseases characterised by excessive immune activation (53, 67). During the COVID-19 pandemic, elevated HO-1 levels were consistently reported in critically ill patients, where the enzyme emerged as both a marker of disease severity and a potential predictor of clinical outcome (53, 127). Moreover, HO-1 expression and activity have been explored as prognostic indicators and therapeutic targets in COVID-19 (128–130).

The pathophysiological processes driving severe COVID-19, including oxidative stress, endothelial dysfunction, and dysregulated cytokine responses, share notable similarities with those observed in malaria (33). In malaria, these processes are further exacerbated by extensive hemolysis and the consequent release of free heme, a potent inducer of HO-1 (35). This mechanistic overlap supports the rationale for extending insights from COVID-19 to malaria and suggests a potential utility of HO-1 as a biomarker in malaria. For instance, HO-1 has shown promise in the following ways:

#### 3.2.1 Severity indicator

Elevated levels of HO-1 (mRNA, protein, or enzymatic activity) have been reported in subjects with severe malaria compared with those with uncomplicated disease or healthy controls (74, 131). This elevation likely reflects the magnitude of hemolysis-induced oxidative stress and inflammatory burden, positioning HO-1 as a candidate indicator of disease severity (35).

#### 3.2.2 Prognostic marker

In severe malaria, particularly cerebral malaria, higher HO-1 expression has been associated with improved clinical outcomes, including reduced mortality and diminished neurological sequelae (66). Conversely, insufficient HO-1 induction in the face of severe infection may signal an impaired cytoprotective response and a poorer prognosis (65, 132, 133).

#### 3.2.3 Therapeutic monitoring

As HO-1-targeted strategies and adjunctive therapies continue to be explored in malaria, monitoring HO-1 levels or activity may serve as a useful tool for assessing therapeutic efficacy and modulating the host response. HO-1 has been proposed as a target for several diseases, including malaria (52, 122, 123).

## 4 Summary of key studies on heme oxygenase-1 in malaria disease

To provide a comprehensive overview of the research landscape on HO-1 in malaria. **Table 1** summarises key *in vitro*, *in vivo*, and clinical studies that have investigated its role. It highlights diverse findings on HO-1 expression, activity, and their impact on parasite burden, host pathology, and clinical outcomes across different *Plasmodium* species and experimental models. Studies have explored various aspects, from the protective effects of HO-1 induction in experimental cerebral malaria models to its association with disease severity in human infections. Insights are provided into the mechanisms by which HO-1 products, such as CO and bilirubin, exert their effects, as well as the potential for HO-1 modulation as a therapeutic strategy. The complex, and often context-dependent, nature of HO-1’s involvement in malaria reinforces its “double-edged sword” character.

## 5 Challenges and future perspectives

The limitations of current malaria diagnostic tools include delayed detection, inability to differentiate between malaria species, and poor prognostic accuracy for severe cases. There is a pressing need for biomarkers that can be measured non-invasively, provide rapid results, and correlate with disease progression. Substantial evidence across malaria, COVID-19, and HIV-associated neurocognitive disorders positions heme oxygenase-1 (HO-1) as a unifying molecular sensor of host stress, immune dysregulation, and tissue injury (105, 134). Rather than serving as a uniform systemic marker, the diagnostic and prognostic value of HO-1 appears to be context-, disease-, and compartment-specific—most informative where oxidative injury and inflammation directly drive pathology. This nuanced behaviour elevates HO-1 beyond a passive biomarker to a dynamic integrator of disease severity, host susceptibility, and therapeutic response.

Despite its promise as a potential diagnostic, prognostic, and therapeutic target, several challenges hinder its clinical application. First, HO-1 is not specific to malaria and is elevated in other hemolytic and inflammatory conditions, underscoring the need for combined biomarker panels to achieve specificity (18, 77). Second, standardised assays for HO-1 measurement are lacking, and variations in assay sensitivity could affect reliability. Third, the cost of developing and deploying HO-1-based diagnostics may be prohibitive in low-resource settings (135).

To accurately interpret the role of HO-1 in malaria, it is important to acknowledge its broader significance in other pathological conditions. HO-1 is widely regarded as a cytoprotective enzyme in diseases driven by oxidative stress and inflammation, including ischemia–reperfusion injury and sepsis, where it limits tissue damage, preserves endothelial function, and modulates excessive immune responses (41, 111, 113). Its antioxidant and anti-inflammatory actions also confer neuroprotection in neurodegenerative and traumatic brain disorders (48), although sustained overexpression may be detrimental in certain contexts (65). In cardiovascular disease, HO-1 mitigates vascular inflammation and oxidative lipid modification (136), whereas in cancer its effects are highly context-dependent, ranging from protection against DNA damage to promotion of tumour progression in established disease (88). The rationale of these findings highlights the pleiotropic and context-specific nature of HO-1, cautioning the need for careful interpretation of its induction in malaria.

Moving forward, it would be encouraging to integrate HO-1 measurements with genetic profiling of HMOX1 variants and clinical phenotyping, as this offers a compelling path toward precision diagnostics and risk stratification, particularly in malaria-endemic and resource-limited settings. Such an approach has the potential to shift HO-1 from a retrospective indicator of damage to a proactive tool for early warning, targeted intervention, and personalised disease management. Future studies should also investigate the role of HO-1 in modulating telomerase dynamics in immune cells of individuals with malaria. HO-1’s upregulation in malaria makes it a candidate for diagnostic assays. Enzyme-linked immunosorbent assays (ELISAs) and point-of-care tests can measure plasma HO-1 levels, providing a rapid, non-invasive diagnostic approach (70). Preliminary studies suggest that HO-1 levels can distinguish malaria from other febrile illnesses, addressing the specificity issues of RDTs (66). Longitudinal studies are needed to establish thresholds for HO-1 levels that predict outcomes, such as cerebral malaria or mortality.

## 6 Conclusion

Heme oxygenase-1 (HO-1) is a central host response enzyme that links heme metabolism, oxidative stress, and immune regulation during malaria infection. Its induction reflects the host’s attempt to counteract hemolysis-driven toxicity and inflammatory injury, processes that are fundamental to malaria pathogenesis and disease severity. Experimental and clinical evidence indicate that appropriately regulated HO-1 activity confers protection against severe manifestations of malaria, including cerebral malaria, severe anaemia, and organ dysfunction, through detoxification of free heme, preservation of endothelial integrity, and modulation of inflammatory responses. However, excessive or prolonged HO-1 expression may compromise antiparasitic immunity, underscoring its context-dependent, dualistic role. Importantly, HO-1 offers clinical value beyond parasite-based diagnostics by providing insight into host tolerance, tissue injury, and immune dysregulation—key determinants of outcome in severe malaria. Integration of HO-1 measurements with parasitological indices, inflammatory markers, and host genetic variation may improve prognostic accuracy and therapeutic monitoring. Nonetheless, challenges such as assay standardisation, disease specificity, and implementation in resource-limited settings remain. Addressing these limitations through longitudinal, multi-biomarker studies will be essential to defining the optimal clinical utility of HO-1. Current evidence positions HO-1 as a promising adjunct diagnostic and prognostic biomarker and therapeutic target, with the potential to enhance precision approaches to malaria management.
